# Prognostic value of the neutrophil-to-lymphocyte ratio in the ARQ 197-215 second-line study for advanced hepatocellular carcinoma

**DOI:** 10.18632/oncotarget.14797

**Published:** 2017-01-22

**Authors:** Nicola Personeni, Laura Giordano, Giovanni Abbadessa, Camillo Porta, Ivan Borbath, Bruno Daniele, Jean-Luc Van Laethem, Hans Van Vlierberghe, Jörg Trojan, Enrico N. De Toni, Antonio Gasbarrini, Monica Lencioni, Maria E. Lamar, Yunxia Wang, Dale Shuster, Brian Schwartz, Armando Santoro, Lorenza Rimassa

**Affiliations:** ^1^ Humanitas Cancer Center, Humanitas Clinical and Research Center, Rozzano, MI, Italy; ^2^ Department of Medical Biotechnology and Translational Medicine, University of Milan, Milan, Italy; ^3^ Clinical Development & Translational Medicine, ArQule, Burlington, MA, United States; ^4^ Fondazione IRCCS Policlinico Universitario San Matteo, Pavia, Italy; ^5^ Cliniques Universitaires Saint-Luc, Brussels, Belgium; ^6^ G. Rummo Hospital, Benevento, Italy; ^7^ Erasme University Hospital, Brussels, Belgium; ^8^ Ghent University Hospital, Gent, Belgium; ^9^ Internal Medicine, J. W. Goethe University Hospital, Frankfurt, Germany; ^10^ Klinikum der Universitaet Muenchen-Grosshadern, Munich, Germany; ^11^ Policlinico Universitario Agostino Gemelli, Rome, Italy; ^12^ Azienda Ospedaliero-Universitaria di Pisa, Pisa, Italy; ^13^ Clinical Development, Daiichi-Sankyo, Edison, NJ, United States; ^14^ Humanitas University, Rozzano, MI, Italy

**Keywords:** hepatocellular carcinoma, neutrophils, neutrophil-to-lymphocyte ratio, tivantinib, MET

## Abstract

The ARQ 197-215 study randomized patients to tivantinib or placebo and pre-specified efficacy analyses indicated the predictive value of MET expression as a marker of benefit from tivantinib in hepatocellular carcinoma (HCC). We aimed to explore the neutrophil-to-lymphocyte ratio (NLR) in 98 ARQ 197-215 patients with available absolute neutrophil count and absolute lymphocyte count at baseline. The cut-off value used to define high versus low NLR was 3.0. In univariate analysis, high NLR was associated with hazard ratio (HR) for overall survival (OS) of 1.58 [95% confidence interval (CI) 1.01; 2.47; *P* <0.046], corresponding to median OS of 5.1 months versus 7.8 months in patients with low NLR (*P* = 0.044). In contrast, time to progression was not significantly affected by NLR (*P* = 0.20). Multivariable model confirmed that both NLR >3 (*P* = 0.03) and presence of vascular invasion (*P* = 0.017) were negatively associated with OS. After adjustment for vascular invasion, NLR independently predicted survival in both the placebo and the tivantinib cohort. For OS, no interaction was detected between NLR status and treatment (*P*_interaction_ = 0.40). Baseline NLR is an independent prognostic biomarker in patients with HCC and compensated liver function who are candidate for second-line treatments.

## INTRODUCTION

Hepatocellular carcinoma (HCC) accounts for nearly 80% of primary liver cancer cases [1] and it is a leading cause of death worldwide [2]. Risk stratification in current clinical trials for advanced HCC is based on clinical, radiologic and biochemical grounds. The underlying relationships that link such commonly used variables to the degree of liver dysfunction, and possible therapeutic interventions, have been further appraised within various prognostic systems [3].

Among patient-related factors that drive prognosis, mounting evidences suggest that cancer-associated inflammation is a determinant of disease progression and survival in several cancer types, including HCC [4, 5]. In particular, the neutrophil-to-lymphocyte ratio (NLR), which is hematological surrogate marker of the systemic inflammatory response, has gained lot of interest in the last decade [6]. The NLR is readily evaluable by peripheral blood tests and specifically refers to the ratio of the absolute neutrophil count (ANC) to absolute lymphocyte count (ALC). In HCC, infiltrating neutrophils may affect disease initiation and progression acting through immunosuppressive mechanisms, which might be mediated either by abnormal chemokine CCL2 production [7] or increased programmed cell death ligand 1 expression [8]. Furthermore, neutrophils promote angiogenesis in peritumoral stroma of HCC via matrix metalloproteinase-9 and vascular endothelial growth factor signaling [9]. On the other hand, the densities of both tumor-infiltrating T and B lymphocytes were shown to be associated with improved survival and decreased tumour aggressiveness in HCC patients [10]. Therefore, an elevated NLR might mirror an imbalance favoring the neutrophil pro-tumorigenic functions at the expenses of a decreased anti-tumor immune surveillance of the host.

These observations are in substantial agreement with a recently published meta-analysis showing that a low baseline NLR is associated with improved overall survival (OS) in HCC patients [11], but the overall quality of the studies included, as acknowledged by the authors, was low. In addition, heterogeneous tumor stages and treatment modalities were considered [11]. Finally, arguments supporting the usefulness of the NLR in advanced HCC patients receiving medical treatments encompass few retrospective series, of which three have been published in full-text form [12–14].

The ARQ 197-215 was a randomized placebo-controlled phase II study testing the MET inhibitor tivantinib for second-line treatment of HCC patients [15]. Although OS was similar in both the tivantinib and the placebo arms of the study, a predefined subgroup analysis highlighted a significant OS benefit from tivantinib in patients with MET-high tumors [15].

Given these premises, the current *post-hoc* analysis was performed within the ARQ 197-215 study and aimed to further evaluate the prognostic impact of the NLR within a prospectively collected, phase II trial dataset of patients with advanced HCC and compensated liver function who were eligible for second-line treatments.

Beyond those variables usually considered for enrollment onto clinical trials, should post-progression survival after first-line therapy be influenced by the NLR, this would represent a novel criterion worth to be considered for the design of future second-line studies in HCC.

## RESULTS

### Patients

A total of 107 patients were enrolled in the ARQ 197-215 study [15]. Nine patients for whom there were no baseline ANC/ALC data were excluded, leaving an eligible NLR population of 98 patients. Sixty-five patients received tivantinib, and 33 received placebo. The prognostic characteristics of patients in the NLR population did not substantially differ from those in the ARQ 197-215 study. OS in the tivantinib and placebo arms was 6.5 and 6.2 months, respectively (*P* = 0.81), and time to progression (TTP) was 1.5 and 1.4 months (*P* = 0.223), respectively. In comparison, OS in the tivantinib and placebo arms of the ARQ 197-215 population [15] was 6.6 and 6.2 months (*P* = 0.63), while TTP was 1.6 and 1.4 months (*P* = 0.04), respectively.

### Baseline NLR and prognostic variables

Table 1 shows baseline characteristics with comparisons between the low NLR and high NLR groups. When patients were grouped by NLR, there were no differences in terms of key baseline characteristics including extrahepatic spread, alpha-fetoprotein (AFP) levels, MET expression levels, macrovascular invasion, treatment arm (tivantinib or placebo). Nevertheless, NLR values were found to be directly associated (*P* <0.001) with PLR values.

### Association between NLR and outcomes in the NLR population

**Figure 1:**
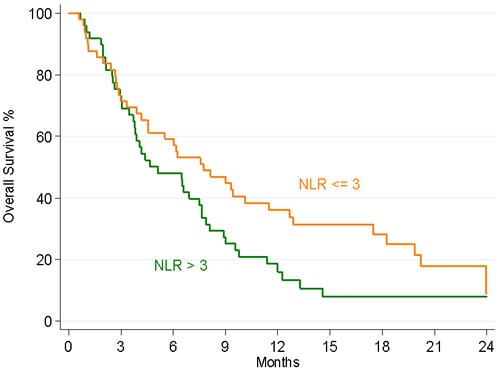
Kaplan–Meier estimates of OS according to NLR levels

With a median follow up of 18.9 months (range 0.6 - 24.8), NLR as a continuous variable was inversely associated with OS [Hazard Ratio (HR) 1.19, 95% confidence interval (CI) 1.07; 1.34; *P* = 0.002]. Consistently, the association between NLR level and OS was confirmed using the median cut-off value of 3.0. The median OS values were 5.1 months in patients with NLR >3 and 7.8 months in patients with NLR ≤3 (*P* = 0.044). Figure 1 displays the Kaplan-Meier curves for OS for the two NLR groups. Compared with the low NLR group, the HR for OS in patients with high baseline NLR was 1.58 (95% CI 1.01; 2.47; *P* <0.046).

**Table 1:** Key baseline characteristics and comparisons between the low (≤3) and high (>3) NLR groups

Group	All patients		NLR ≤3 (*N* = 49)		NLR >3 (*N* = 49)		*P* value
	*N*	%	*N*	%	*N*	%	
**Treatment**							
placebo	33	33.7	18	36.7	15	30.6	0.521
tivantinib	65	66.7	31	63.3	34	69.4	
**Macrovascular invasion**							
No	64	65.3	33	51.6	31	48.4	0.671
Yes	34	34.7	16	47.1	18	52.9	
**Distant metastases**							
No	33	33.7	18	54.5	15	45.5	0.521
Yes	65	66.3	31	47.7	34	52.3	
**Baseline alpha-fetoprotein**							
≤ median	47	48.0	23	48.9	24	48.9	0.837
> median	47	48.0	24	51.1	23	51.1	
missing	4	4.0					
MET expression							
Low	35	35.7	19	54.3	16	45.7	0.552
High	36	36.7	17	47.2	19	52,8	

**Figure 2:**
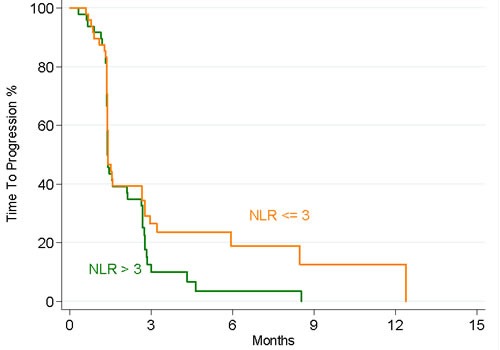
Kaplan–Meier estimates of TTP according to NLR levels

Also, when the NLR was evaluated as a continuous trait, the HR for TTP was 1.07 (95% CI 0.96; 1.19; *P* = 0.20). The corresponding median TTP value in both high and low NLR groups was 1.4 months (*P* = 0.10; Figure 2).

### Long-term survival

There were 22 long-term survivors, defined as patients surviving beyond 12 months. Of these, 16 (72.7%) had low NLR compared with 6 (27.2%) with high NLR. This equated to 32.6% (16 of 49) of all patients with a low NLR achieving survival beyond 12 months compared with only 12.2% (6 of 49) of all patients with high NLR. The Chi-squared test confirmed an association between low baseline NLR and long-term survival (*P* = 0.015).

### Multivariable analysis

In univariate analyses, NLR and vascular invasion were significantly associated with OS. In contrast, AFP median levels, MET expression levels, treatment group, and extrahepatic spread were not (data not shown).

Additionally, using the median cut-off value of 167, a PLR ≤167 was associated with a median OS of 9.0 months compared to 4.5 months in patients with PLR >167 (HR 1.60, 95% CI 1.02; 2.50, *P* = 0.040). However, due to the strong association with NLR and because PLR analyses are beyond the scope of the present study, in order to avoid collinearity of single variables and redundancy, further analyses were undertaken without the PLR. The Cox model confirmed that only NLR and vascular invasion independently predicted OS. In this model, NLR >3 resulted in poorer survival (HR 1.65; 95% CI 1.05; 2.59; *P* = 0.030), as well as macrovascular invasion (HR 1.74; 95% CI 1.10; 2.75; *P* = 0.017).

### Association between NLR and outcomes according to treatment arm

In the tivantinib arm, median OS values were 6.5 months in patients with NLR >3 and 7.6 months in patients with NLR ≤3 (*P* = 0.231). In the placebo arm, median OS values were 4.2 months in patients with NLR >3 and 7.9 months in patients with NLR ≤3 (*P* = 0.048). Modeling NLR as a continuous variable increased the statistical significance of the relationship existing between increased NLR and risk of death in both tivantinib (HR 1.16, 95% CI 1.00; 1.30, *P* = 0.049) and placebo arms (HR 1.30, 95% CI 1.08; 1.57, *P* = 0.006). In other words, these findings confirm a concrete risk of death, which gradually increases with increasing NLR, but it is not specific to NLR ≤3 versus >3. In a Cox model, after adjustment for vascular invasion, these results remained significant in both tivantinib (HR 1.18, 95% CI 1.01; 1.38, *P* = 0.042) and placebo arms (HR 1.32, 95% CI 1.09; 1.61, *P* = 0.004). On the other hand, consistent with results observed in the entire NLR population, the associations between NLR (either as continuous or categorical variable) and TTP within both treatment arms of the trial were not significant (data not shown).

### Predictive analyses

Among patients with NLR ≤3, the HR for tivantinib effect on OS was 1.2 (95% CI 0.61; 2.38; *P* = 0.594), compared with 0.65 (95% CI 0.34; 1.25; *P* = 0.199) in patients with NLR >3. However, the *P* value for the interaction between NLR value and treatment was 0.40, thus ruling out a differential effect of tivantinib between the two NLR groups. These results confirm that the NLR value does not have a predictive role in relation to treatment selection. In contrast, also in the current NLR population, MET expression confirmed its predictive role as a biomarker of tivantinib efficacy in HCC (*P*_interaction_ = 0.039), as already shown in a previous investigation [16].

### Changes in NLR and clinical outcomes

Neutropenia was among the most frequent tivantinib-related adverse events, generally occurring within the first 30 days of treatment [15]. Included in this analysis were patients for which NLR was available at D1C1 and at day 1 of cycle 2 (D1C2), which was scheduled 28 days after D1C1. Median variation of ANC (all-patient cohort) during the first cycle of treatment with tivantinib or placebo was -9.0% (range -73.4%; +206.0%). In patients receiving tivantinib, the mean variation was -11.7% (95% CI -22%; -1.3%) and was statistically significantly different from the mean neutrophil variation observed in patients receiving placebo (+10.8%, 95% CI -8.8%; 30.5%; *P*_t-test_ = 0.045). When the neutrophil variation was evaluated as a continuous variable, no survival differences were observed in univariate analysis (*P* = 0.2), nor after adjustment for treatment assignment (P = 0.3). When the two pre-therapy NLR groups (NLR ≥3 or <3) were further dichotomized according to NLR results at D1C2, the four pre-therapy and post-therapy NLR groups were: low-low (pre-therapy NLR <3 and post-therapy NLR <3) in 31 (38.2%) cases; high-low (pre-therapy NLR ≥3 and post-therapy NLR <3) in 12 (14.8%); high-high (pre-therapy NLR ≥3 and post-therapy NLR ≥3) in 29 (35.8%); low-high (pre-therapy NLR <3 and post-therapy NLR ≥3) in 9 (11.1%). Given the limited number of patients experiencing a NLR variation from high to low NLR categories or vice versa, and the few OS events observed among these patients, further survival analyses according to this model were inconclusive (data not shown).

## DISCUSSION

In the present study, NLR is an independent predictor of OS in a homogeneous cohort of HCC patients, having Child-Pugh A or no liver cirrhosis, who are candidate for second-line treatment. In such patients, the survival differences attributed to NLR status are substantial, particularly in light of the survival outcomes reported in the ARQ 197-215 study [15]. Furthermore, considering the results of the recently reported RESORCE trial [17], we believe that in a second-line setting these survival differences are of clinical interest.

The discriminatory effect of NLR was observed also within both treatment arms of the ARQ 197-215 study, namely tivantinib and placebo. Of note, this indicate that the prognostic value of NLR is independent of a potential key confounder represented by treatment allocation. Whereas tivantinib was shown to significantly prolong TTP [15], this genuine radiological endpoint did not appear to be affected by the NLR. Additionally, the survival analysis according to treatment arm suggested that the OS benefit deriving from tivantinib over placebo could be greater in patients with NLR >3, compared to patients with NLR ≤3. However, these results have to be cautiously interpreted due to the small number of patients considered. Furthermore, the interaction test between NLR and treatment did not reach statistical significance. At present, MET expression is the only available biomarker predicting tivantinib efficacy in advanced HCC [16], and these findings remained confirmed even within the slightly smaller cohort of patients with available MET results considered in the current report. In contrast to a previous investigation [18], a high NLR was not associated with other negative prognostic factors traditionally considered in the setting of advanced HCC, such as presence of macrovascular invasion, high AFP levels, or extrahepatic metastases. The only remarkable association was observed with respect to the PLR, which is another marker of systemic inflammatory response. Present data on the relationship between PLR and survival in advanced HCC are scarce and rather conflicting [13, 19], whereas overall data on NLR appear to be more robust. Recently, platelets counts, in addition to ANC and ALC, have been incorporated within a systemic immune-inflammation index (SII) that allowed to discriminate prognosis in a retrospective cohort of 56 HCC patients treated with sorafenib [14]. Similar findings on the prognostic value of the SII were described also in the context of less advanced disease stages [20]. It is still unclear which one, between SII and NLR, could be the most useful prognosticator in advanced HCC.

Interestingly, in small-cell lung cancer it has been reported that post-chemotherapy decline of NLR from high to low is associated with improved survival [21]. Neutropenia was among the most frequent adverse events associated with tivantinib [15]. Accordingly, mean ANC variations at the end of the first cycle of treatment were significantly different in patients receiving tivantinib or placebo. However, although 12 patients experienced a decline of NLR from high into low category, their OS was not significantly different from patients with persistently high NLR.

Our results extend previous retrospective observations in the frame of a prospective, placebo-controlled randomized trial which allows to control for potential key confounders. Of note, the NLR can also be affected by transient infection or drug treatments, but in this study values were recorded in patients with no active infection and prior to the second-line treatment. Additionally, blood counts were performed at a central lab, thereby minimizing preanalytical/analytical biases related to assessments done according to diverse instrumental parameters and diverse reference ranges. To our knowledge, this report represents one of the largest individual analyses evaluating NLR in an advanced/metastatic HCC population, homogeneous for prior treatments and clinical baseline characteristics.

These are among the main strengths of the current investigation. Other determinants of postprogression survival have been recently reported in patients with compensated liver function who permanently discontinue sorafenib, including patterns of radiological progression [22] and reasons for sorafenib discontinuation [22, 23]. These were not fully acknowledged at the time of the ARQ 197-215 protocol writing, and therefore they could not be captured in the present analysis. This is a limitation inherent to this and other trials where protocols were written before these data were published.

A number of cut-off values to categorize NLR have been reported in the setting of several cancer types. The heterogeneity of such cut-offs could make conclusions on the clinical utility of NLR and comparisons between studies somehow difficult. However, results of the current study overall indicate that the 3.0 cut-off value, which is adopted in most studies, is easily reproducible, even outside the training sets where it was first generated. Clearly, for the development of the NLR as an useful tool for prognosis, a consensus needs to be achieved on the definitions of ‘high’ versus ‘low’ NLR.

In conclusion, on the basis of the current study, low cost, reproducibility, and easy evaluation of a full blood count render the NLR worth of consideration as a stratification factor in novel clinical trials for HCC.

Validation of the NLR in the larger, ongoing ARQ 197-A-U303 study (NCT01755767) is warranted.

## materials and METHODS

### Patients and trial design

Trial design, eligibility criteria and results of the ARQ 197-215 were previously reported [15]. The primary objective of the ARQ 197-215 study was to compare the effect of tivantinib versus placebo on TTP. Eligible patients were enrolled from 23 centers in Italy, Belgium, Germany, Canada, and USA. Briefly, they had to have Eastern Cooperative Oncology Group performance status ≤1, Child-Pugh A or no liver cirrhosis, adequate bone marrow, liver, and renal function, histologically or cytologically confirmed, unresectable, advanced-stage HCC. The eligible population for the current exploratory analysis (herein referred to as NLR population) included all ARQ 197-215 participants with available white blood cell, ANC and ALC data at the day 1 of the first cycle. The NLR was calculated using the standard formula: NLR = ANC/ALC. Analogously, the platelet-to-lymphocyte ratio (PLR) was calculated as a ratio between the absolute platelet count to the ALC. Laboratory assessments including hematology at day 1 of each treatment cycle were performed at a central lab.

### Statistical methods

The objective of the present study was to examine the relationship between NLR and prognosis. NLR was first evaluated as a continuous variable: should a statistically significant association be observed, then the NLR was categorized. Patients were therefore grouped into ‘high’ and ‘low’ NLR populations based upon a cut-off value of 3.0, which was determined on the basis of prior publications in HCC [14] and other solid tumors [24]. The 3.0 cut-off also corresponds to the median NLR value observed in the current NLR population (range 0.8-12.8), which can be used in the absence of further clinical evidences on a cut-off value to differentiate low and high baseline NLR levels. TTP was calculated from the start of first-line treatment until disease progression. OS was calculated from the start of first-line treatment until death or last follow-up. Kaplan-Meier method was used to estimate and plot survival curves and differences between groups were tested by the log rank test. Variables which were statistically significant in the univariate analysis were entered into a multivariable Cox proportional hazards model to assess the prognostic value of baseline NLR taking into account the effect of confounding factors. Interaction between treatment group and NLR was assessed to verify whether the treatment effect differed between the NLR groups.

Data were summarized as frequencies, proportions and differences between groups were tested by the Chi-squared test for categorical data. Continuous variables were described as median and range, the Pearson correlation coefficient was used to test for correlation, differences between groups were evaluated comparing means by the *t*-test. All analyses were done using the Stata version 13 statistical package. *P* <0.05 was considered significant.

## Abbreviations

HCC: Hepatocellular carcinoma
AFP: Alpha-fetoprotein
NLR: neutrophil-to-lymphocyte ratio
ANC: absolute neutrophil counts
ALC: absolute lymphocyte counts
OS: overall survival
TTP: time to progression
PLR: platelet-to-lymphocyte ratio
HR: Hazard Ratio
CI: confidence interval
SII: systemic immune-inflammation index
